# First Evidence and Predictions of *Plasmodium* Transmission in Alaskan Bird Populations

**DOI:** 10.1371/journal.pone.0044729

**Published:** 2012-09-19

**Authors:** Claire Loiseau, Ryan J. Harrigan, Anthony J. Cornel, Sue L. Guers, Molly Dodge, Timothy Marzec, Jenny S. Carlson, Bruce Seppi, Ravinder N. M. Sehgal

**Affiliations:** 1 Department of Biology, San Francisco State University, San Francisco, California, United States of America; 2 Center for Tropical Research, Institute of the Environment and Sustainability, University of California Los Angeles, Los Angeles, California, United States of America; 3 Mosquito Control and Research Laboratory, Department of Entomology, University of California Davis, Parlier, California, United States of America; 4 Alaska Bird Observatory, Fairbanks, Alaska, United States of America; 5 Bureau of Land Management, Anchorage Field Office, Anchorage, Alaska, United States of America; Université Pierre et Marie Curie, France

## Abstract

The unprecedented rate of change in the Arctic climate is expected to have major impacts on the emergence of infectious diseases and host susceptibility to these diseases. It is predicted that malaria parasites will spread to both higher altitudes and latitudes with global warming. Here we show for the first time that avian *Plasmodium* transmission occurs in the North American Arctic. Over a latitudinal gradient in Alaska, from 61°N to 67°N, we collected blood samples of resident and migratory bird species. We found both residents and hatch year birds infected with *Plasmodium* as far north as 64°N, providing clear evidence that malaria transmission occurs in these climates. Based on our empirical data, we make the first projections of the habitat suitability for *Plasmodium* under a future-warming scenario in Alaska. These findings raise new concerns about the spread of malaria to naïve host populations.

## Introduction

In the Arctic, the threats of global warming are imminent, with temperatures increasing at almost twice the average global rate [1], [2]. Sea ice has thinned, and its extent has been greatly reduced, as has the terrestrial permafrost [3]. These changes have already resulted in vegetation community shifts, with a transition from grasses to shrubs [4]. Climate and habitat alterations will also likely impact the dynamics of infectious diseases [5], [6]. Global changes have modified the timing of parasite life cycles [7], enhanced transmission and outbreaks [8], and will have significant consequences on the expansion of the geographical northern range of parasites, which is a considerable concern given the likely invasion by parasites into regions inhabited by naïve host populations [7], [9], [10].

Malaria parasites are among the most intensively investigated vector-borne pathogens worldwide [11]. Under a scenario of rapid global change, it is crucial to understand how the modification of environmental conditions will affect the spatial distributions of *Plasmodium* species. Past empirical and modeling studies have provided some evidence for the shifting in patterns of abundance and diversity of vector-borne parasites with climate change [12], [13], [14]. Diurnal temperature variation has recently been identified as an important factor for the development of *Plasmodium* parasites [15]. In addition, altitude has shown to have an effect on avian malaria presence due to thermal constraints on the sporogonic development of the parasite in the vector [16]. Precipitation and temperature are not only associated with the parasite development in the vector, but also with the larval abundance and vector development itself that could play a significant role in the spread of malaria parasites [17], [18].

With global climate change, it has been predicted that malaria will likely spread to any area that presents suitable environmental conditions for the parasite, including those at higher elevations and more northerly latitudes [14]. Whereas human malaria is also expected to respond to climate change, it has been exceedingly complex to develop predictive models for these systems, partly due to interactions with socioeconomic factors [19], [20]. Thus, avian malaria serves as an ideal natural model system for the study of the effects of environmental change on parasite distribution patterns.

In Arctic regions, migratory Fennoscandian bluethroat (*Luscinia svecica*) in Sweden were found to be infected with a *Plasmodium* lineage [21], however samples were collected only from adults, raising the possibility that transmission occurred outside of the Arctic Circle. Similarly, none of the investigations in North America have demonstrated the transmission of *Plasmodium* parasites in northern latitudes (61°N to 67°N), since they did not screen residents nor hatch year birds [22], [23], [24]. To our knowledge, two studies have demonstrated that malaria transmission can occur in the Arctic, where two resident species (the Siberian tit, *Poecile cinctus* and the House sparrow, *Passer domesticus*) were found infected with *Plasmodium* parasites in Finland and Norway [25], [26]. It is important to note though, that similar latitudes in North America and Europe do not experience similar environmental conditions. While it is evident that temperatures in the northern latitudes have risen over the last half of the 20th century, it is still unknown how these shifts in temperature boundaries have affected the spread of avian malaria parasites.

Over a latitudinal gradient in Alaska, we determined the prevalence and diversity of avian *Plasmodium*, using molecular screening of migratory and resident birds at three locations: Anchorage (61°N), Fairbanks (64°N), and Coldfoot (67°N). We characterized the environmental conditions at each site using a suite of bioclimatic and topography metrics. Based on our model of the empirical data of this disease in the New World Arctic, we produced a first map of the current *Plasmodium* transmission in Alaska, and a predictive map under a future-warming scenario. We provide clear evidence that malaria transmission can occur in these climates, and discuss concerns about the effects of climate change on malaria transmission to new host populations in the northern Arctic.

## Materials and Methods

### Samples Collection

Samples were collected at three latitudes in 2011: Anchorage (61°N), Fairbanks (64°N) and Coldfoot (67°N). We sampled 3, 1 and 4 sites respectively, at these latitudes (Table 1). In total, 676 individuals were captured representing 32 bird species (10 resident species and 22 migratory species; Table S2). Birds were caught using mist-nets and were banded and aged using presence or absence of cloacal protuberance/brood patch or by skull pneumatization [27]. Blood samples were collected from the brachial vein and stored in lysis buffer (10 mM Tris-HCL pH 8.0, 100 mM EDTA, 2% SDS).

**Table 1:** GPS coordinates, altitude, number of infected resident birds (*N* infected/*N* total), numbers of migratory hatch year (HY) and after hatch year (AHY) individuals infected with *Plasmodium* spp. are given by location.

Location	Site	Latitude (N)	Longitude (W)	Alt	N _Resident_	N _Migratory_ HY	N _Migratory_ AHY
Anchorage	Potter Marsh	61°04′17.62″	149°48′26.20″	55	1/2	0/0	0/3
	South Bivouac	61°09′19.25″	149°44′45.88″	150	0/10	0/0	3/44
	Campbell Creek	61°09′50.00″	149°46′11.85″	104	2/35	2/113	9/65
Fairbanks	Creamers Field	64°52′03.20″	147°44′52.16″	134	4/76	1/68	11/103
Coldfoot	Rosie Creek S	67°10′52.06″	150°18′19.06″	427	0/16	0/7	1/25
	Rosie Creek N	67°11′44.66″	150°16′45.96″	384	0/4	0/4	0/22
	Slate Creek	67°14′06.23″	150°07′52.90″	345	0/11	0/0	7/41
	Marion Creek	67°19′07.35″	150°09′23.64″	330	0/6	0/0	8/22
Total					7/160	3/192	39/325

Residents and hatch year migratory birds were infected by one lineage *Plasmodium* P43 whereas after hatch year migratory birds were infected by six lineages of *Plasmodium* spp (see Supporting Information).

### Molecular Analysis

DNA was extracted from whole blood from birds following a DNeasy kit protocol (Qiagen, Valencia, California). For *Plasmodium* detection, we used a nested PCR to amplify a fragment of cytochrome *b* with the primers HAEMF/HAEMR2 - HAEMNF/HAEMNR2 [28]. We used *AccuPower*® HotStart PCR PreMix manufactured by Bioneer. Primers were mixed with purified water and added to the PCR tubes to make a total volume of 20 µl including the DNA template. The PCR products were run out on a 2% agarose gel using 1×TBE, and visualized by an ethidium bromide stain under ultraviolet light to check for positive infections. PCR products from birds infected with *Plasmodium* were purified using ExoSap (following the manufacturer’s instructions, USB Corporation, Cleveland, Ohio) and sent out for Bi-directional sequencing to Elim Biopharmaceuticals Inc., Hayward, CA. Lineages found in this study are listed in Table S2 with their Genbank accession numbers. *Plasmodium* P43 shows only one base pair of difference with *Plasmodium circumflexum* (Genbank accession number: JN164734 (TURDUS-1)) and represents inter-species genetic variation (0.2% of genetic difference).

### Predictive Map of *Plasmodium* Transmission

Each location differed in terms of habitat and climate (Supporting Information S1). These environmental characteristics of each site were determined using a set of environmental variables that included bioclimatic metrics at 1 km from the WorldClim data set [29], http://www.worldclim.org) (Supporting Information S1). We constructed classification trees using a random forest model (RandomForest) [30] with R software [31], with bioclimate and elevation variables (extracted for each site) as predictors, and site location (three separate classes) as a response variable. We used these classification models to predict the range of transmission occurrence of *Plasmodium* across the state of Alaska. These models had a 90% correct classification rate. We then randomly created 10,000 sites within Alaska and predicted whether these sites were suitable for transmission or not according to environmental conditions. Values were plotted and interval points between were interpolated using ordinary Kriging within the ArcMap framework (Environmental Systems Resource Institute, ArcMap 10.0 ESRI, Redlands, California). Future environmental conditions (the same 7 variables that were used for classification and current environmental predictions) were obtained from the Worldclim group and downloaded at 1 km resolution for North America, under the SRES A1 scenario for the year 2080 [32]. Predictions at each of the same 10,000 random points used to construct current spatial predictions were made, and ordinary Kriging [33] was performed to construct a continuous projected surface across Alaska.

## Results

### Parasite Transmission

From 676 individuals, we found 7.2% of infected individuals with *Plasmodium* parasites and detected 7 distinct lineages of *Plasmodium* spp. (Table S2). To be certain that *Plasmodium* transmission occurs at a location, we examined for the presence of infection in resident birds and hatch year birds. We found, by PCR and sequencing, only one parasite, *Plasmodium circumflexum* (*Plasmodium* P43) capable of completing its life cycle in Alaska. Three residents Boreal chickadees (*Poecile hudsonicus*) and two hatch year individuals (Varied thrush, *Zoothera naevia* and Fox sparrow, *Passerella iliaca*) were infected with this lineage in Anchorage (61°N) and four Black-capped chickadees (*Poecile atricapillus*) and a hatch year Myrtle warbler (*Dendroica coronata coronata*) in Fairbanks (64°N) but none were infected in Coldfoot (67°N).

### Predictive Map of *Plasmodium* Transmission

**Figure 1:**
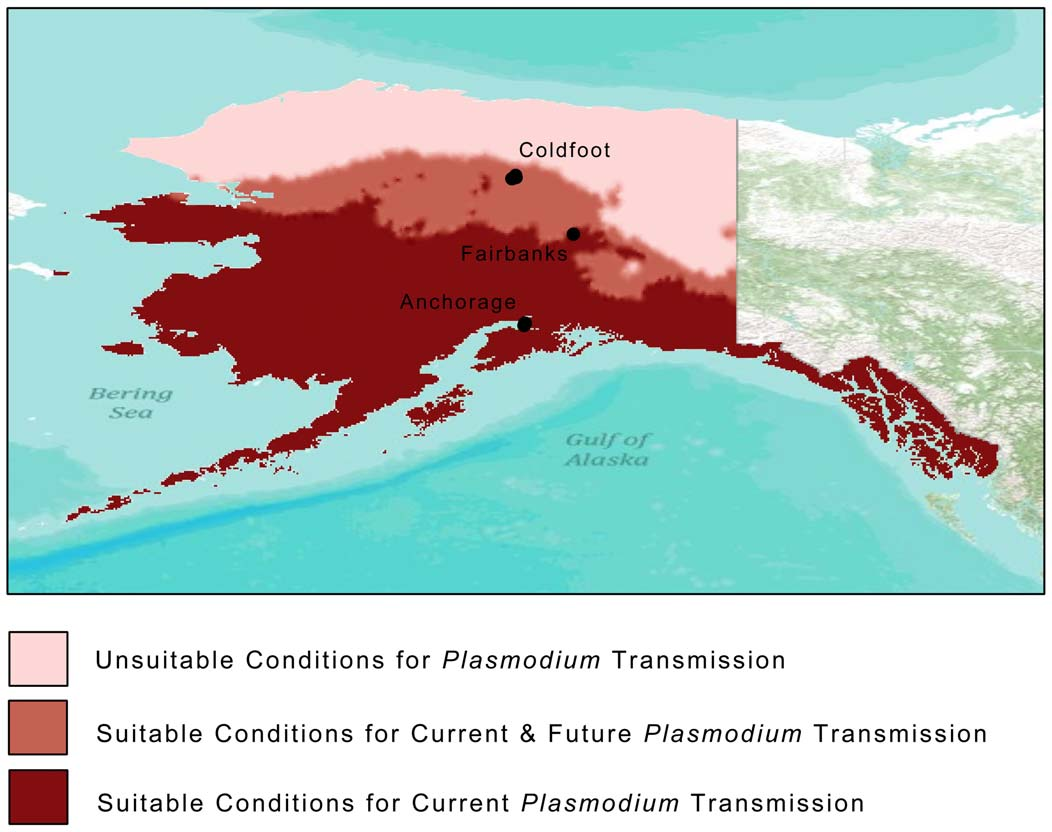
Predictive map of suitable conditions of current and future *Plasmodium* transmission in Alaska with location of sampling sites in each of the three latitudes: Anchorage, Fairbanks and Coldfoot. Map created in ArcMap 10 (Environmental Systems Resource Institute, ArcMap 10.0 ESRI, Redlands, California).

Based on our empirical data, we developed a predictive map of *Plasmodium* transmission (Fig. 1). Bioclimatic variables showed a significant difference in climate patterns among our locations. Under a random forest classification model, climate variables correctly classified 87.5% of the sites. The variables most important in determining these classifications were annual precipitation (BIO12), temperature seasonality (BIO4), precipitation seasonality (BIO15) and minimum temperature of the coldest month (BIO6).

In order to compare our results to the only other identification of *Plasmodium* transmission at extremely northern latitudes, we ran the same classification regression including the site in Finland (Kuusamo, 66°N 29°E), where transmission of *Plasmodium* has occurred [25]. Even though the latitude was within the Arctic Circle (66°N), this site in Finland was classified with the sites in Anchorage. In other words, 61°N sites in Anchorage and the Finland site at 66°N are indistinguishable with regards to this set of bioclimatic variables.

Using these classification regressions, we were able to determine the habitat suitability for *Plasmodium* transmission even at unsampled locations. Roughly south of a thin latitudinal band at 64°N, we found conditions suitable for transmission, including temperature seasonality and annual precipitation, based on where we observed transmission to have occurred. Further north of this band, we found current conditions that would make transmission unlikely based on our observation of only adult migrants with *Plasmodium* at these latitudes. When projecting these classification regressions to the same locations under a future-warming scenario (IPCC 4^th^ Assesment A1 scenario for the year 2080), we found a northerly shift of the cutoff band for conditions suitable for *Plasmodium* transmission (Fig. 1). Although these current and future projections are based only on the eight sites at which we sampled, our overall low classification error (12.5%) as well as our extensive sampling at each location (hatch year and resident birds: n = 150, 144, 48, respectively) provide confidence in these projections, and allow us to use them as a testable spatial distribution for verification in future ground-truthing efforts.

## Discussion

We have shown here, for the first time, that the *Plasmodium* parasites that cause avian malaria are able to complete their transmission cycle in the North American Arctic up to, but not above, 64°N. Our results also provide empirical evidence that local hosts in the north of Alaska may be exposed to new parasites with impending global warming since variation of diurnal or seasonal temperature can lead to a rapid change on the host-vector-parasite interaction [34]. Here, the main bioclimatic metrics that distinguished the sites at the three different latitudes were the annual precipitation, as well as the temperature and precipitation seasonality. Therefore if we consider Anchorage and Fairbanks to have suitable conditions for the *Plasmodium* life cycle completion, other areas with high annual precipitation but mild precipitation and temperature seasonality would be predicted to also be suitable for *Plasmodium* development. This finding is in accordance with a recent study, carried out in West and Central Africa, in which the authors determined that seasonality in temperature was a key predictor for the presence of avian malaria lineages (*Plasmodium* spp.) [35]. Now that Alaska has been identified as an area with suitable climatic conditions for the completion of the avian malaria transmission cycle, a long-term monitoring of *Plasmodium* occurrence as it relate to climate variation in this region is essential.

The parasite *Plasmodium circumflexum* P43 is a highly generalist parasite able to infect individuals across families and even across orders of birds. This lineage has been found previously in four bird species: the Common Rosefinch (*Carpodacus erythrinus*) in South Korea, the Greater Scaup (*Aythya marila*), the Pacific Golden Plover (*Pluvialis fulva*) and the Common Yellowthroat (*Geothlypis trichas*) in the United States, and additionally, in six migratory species in this study (Table S2). This malaria species is a likely cold-tolerant species capable of northward movement. Habitats subject to highly seasonally variable environmental conditions, as exemplified by the Arctic regions, are expected to harbor generalist parasites [36]. Due to dramatic temperature changes across seasons, parasites in the Arctic have a small time window to be effectively transmitted; therefore the optimal strategy is to increase the range of potential hosts [37].

At our most northerly sampled latitudes above the Arctic Circle, at sites near Coldfoot, we also found adult Swainson's Thrush (*Catharus ustulatus*) infected with *Plasmodium* P43, confirming that even at this latitude, birds are able to carry the parasite and serve as hosts of a complete transmission cycle. Follow up sampling conducted seasonally in Coldfoot and other Alaskan locations should be done to better determine *Plasmodium* P43 distributions, and more importantly, what pathogenic and fitness effects it has on Arctic birds. Our concern is warranted in this case, as it is predicted that generalist parasites are the most capable of adapting to new hosts and new areas, and are likely to have devastating impacts on immunologically naïve bird populations. Mitigation of the impacts of this disease in northern latitudes is essential to help prevent the severe consequences malaria has previously had in more southern latitudes, for example, the introduction of *Plasmodium relictum* GRW4 and its competent vector *Culex quinquefasciatus* to Hawaii [38].

It has previuously been demonstrated that *Culiseta morsitans* is a competent vector in New Brunswick for avian *Plasmodium* [39]. This species is considered very rare in Alaska, however *Culiseta alaskaensis* (Ludlow) is one of the main mosquitoes present in a large part of the Alaska state [40]. To fully test our predictions presented here, it will be important during our long-term monitoring to determine the actual vector of avian *Plasmodium,* perform experimental work with local infected birds, and examine the requirement for the parasite to complete its life cycle. The resulting information would be invaluable in assessing effects of global changes on the spread of malaria parasites in the Arctic regions.

We do not discount here the numerous other variables that may affect transmission, such as the larval development, life cycles, and environmental conditions of mosquito vectors [17], [18], nor the geographical range shifts of avian hosts with climate change [41]. However, given that many if not all resident Arctic populations are likely immunologically naïve, and that millions of migratory birds occupy these habitats simultaneously during short breeding seasons, the fitness of individuals and the population dynamics of each region should be wholly considered. Understanding the current and future spatial distributions of malaria in Arctic regions should now be at the forefront for protecting Arctic wildlife populations.

## Supporting Information

Table S1 Bioclimatic and habitat variables per location.
(PDF)

Table S2 List and number of individuals captured (resident (R) vs. migratory (M)) and Plasmodium lineages (Genbank accession number) found by species and location.
(PDF)

Supporting Information S1 (PDF)
