# Supplementary material for: First Evidence and Predictions of Plasmodium Transmission in Alaskan Bird Populations
Source: PLoS One. 2012 Sep 19;7(9):e44729. doi: 10.1371/journal.pone.0044729 (PMC3446979; doi:10.1371/journal.pone.0044729)
Supplement: Table S1 — Bioclimatic and habitat variables per location. (PDF) [file pone.0044729.s001.pdf]

Table S1
Bioclimatic and habitat variables per location.

Location	Site	Latitude (N)	Longitude (W)	BIO1	BIO4	BIO5	BIO6	BIO12	BIO15	BIO19	NDVI	QSCAT	Tree cover
Anchorage	Potter Marsh	61°04’17.62”	149°48’26.20”	2.1	8671	18.4	-13.8	490	45	104	8286	-1149	60
Anchorage	South Bivouac	61°09’19.25”	149°44’45.88”	1.6	8716	18.1	-14.4	479	44	101	8699	-912	72
Anchorage	Campbell Creek	61°09’50.00”	149°46’11.85”	1.8	8837	18.3	-14.5	464	45	96	8815	-864	62
Fairbanks	Creamers Field	64°52’03.20”	147°44’52.16”	-2.9	14410	22.3	-27.3	318	47	58	8462	-968	39
Coldfoot	Rosie Creek S	67°10’52.06”	150°18’19.06”	-7.5	14672	19.9	-31.8	295	58	45	7688	-1107	0
Coldfoot	Rosie Creek N	67°11’44.66”	150°16’45.96”	-7.4	14715	20.1	-31.7	294	58	45	7966	-1107	14
Coldfoot	Slate Creek	67°14’06.23”	150°07’52.90”	-7.3	14763	20.3	-31.8	289	57	43	7854	-1107	35
Coldfoot	Marion Creek	67°19’07.35”	150°09’23.64”	-7.4	14771	20.2	-31.9	285	58	43	77827	-1085	23
