# Supplementary material for: First Evidence and Predictions of Plasmodium Transmission in Alaskan Bird Populations
Source: PLoS One. 2012 Sep 19;7(9):e44729. doi: 10.1371/journal.pone.0044729 (PMC3446979; doi:10.1371/journal.pone.0044729)
Supplement: Table S2 — List and number of individuals captured (resident (R) vs. migratory (M)) and Plasmodium lineages (Genbank accession number) found by species and location. (PDF) [file pone.0044729.s002.pdf]

Table S2
List and number of individuals captured (resident (R) vs. migratory (M)) and *Plasmodium* lineages (Genbank accession number) found by species and location.

Species	N	R/M	N_{inf}	Location	Lineage
Alder flycatcher	7	M	1	Coldfoot	*Plasmodium* P48 (DQ659586)
American robin	17	M	1	Anchorage	*Plasmodium* P43 (DQ839065)
Black-capped chickadee	46	R	4	Fairbanks	*Plasmodium* P43
Blackpoll warbler	3	M	-		
Boreal chickadee	13	R	3	Anchorage	*Plasmodium* P43
Brown creeper	2	R	-		
Common redpoll	78	R	-		
Downy woodpecker	2	R	-		
Fox sparrow	11	M	1	Anchorage	*Plasmodium* P43
Golden-crowned kinglet	2	M	-		
Golden-crowned sparrow	2	M	-		
Gray jay	9	R	-		
Hermit thrush	46	M	8	Anchorage	*Plasmodium* P43
Lincoln sparrow	3	M	-		
Myrtle warbler	123	M	3	Fairbanks	*Plasmodium* P43
			1	Fairbanks	*Plasmodium* AK4 (JQ026527)
			1	Coldfoot	*Plasmodium* P1 (AY377128)
Northern waterthrush	8	M	1	Coldfoot	*Plasmodium* 54 (AF465554)
Orange crowned warbler	10	M	1	Coldfoot	*Plasmodium* P48
Olive sided flycatcher	1	M	-		
Pine grosbeak	1	R	-		
Pine siskin	4	R	-		
Red-breasted nuthatch	2	R	-		
Ruby crowned kinglet	1	M	1	Coldfoot	*Plasmodium* P48
Slate-colored junco	89	M	1	Coldfoot	*Plasmodium* P48
Swainson’s thrush	149	M	1	Anchorage	*Plasmodium* P48
			1	Anchorage	*Plasmodium* AK3 (JQ026526)
			3	Fairbanks	*Plasmodium* P48
			4	Fairbanks	*Plasmodium* P43
			1	Fairbanks	*Plasmodium* AK3
			1	Coldfoot	*Plasmodium* AK2 (JQ026525)
			6	Coldfoot	*Plasmodium* P48
			3	Coldfoot	*Plasmodium* P43
Varied thrush	11	M	2	Anchorage	*Plasmodium* P43
White crowned sparrow	21	M	-		
White winged crossbill	2	R	-		
Wilson’s warbler	9	M	-		
Yellow warbler	4	M	-		
