# Supplementary material for: First Evidence and Predictions of Plasmodium Transmission in Alaskan Bird Populations
Source: PLoS One. 2012 Sep 19;7(9):e44729. doi: 10.1371/journal.pone.0044729 (PMC3446979; doi:10.1371/journal.pone.0044729)
Supplement: Supporting Information S1 — (PDF) [file pone.0044729.s003.pdf]

Supplemental Information

Bioclimatic and habitat variables description. We used a set of moderately high-resolution climate and satellite remote sensing variables to characterize the habitat differences among our sampling areas. Variables were re-aggregated from their native resolutions to 5 km resolution (Table S1).

1- Bioclimatic variables

We used 7 bioclimatic variables (representing both temperature and precipitation, while excluding those that showed high degrees of autocorrelation) from the WorldClim database[1] which are 50-year averages (1950-2000) of annual means, seasonal extremes and degrees of seasonality in temperature and precipitation, and represent biologically meaningful variables for characterizing species range[2,3]: (http://biogeo.berkeley.edu/worldclim/bioclim.htm)

BIO1 = Annual Mean Temperature (degree Celsius, °C)

BIO4 = Temperature Seasonality (standard deviation *100)

BIO5 = Max Temperature of Warmest Month (degree Celsius, °C)

BIO6 = Min Temperature of Coldest Month (degree Celsius, °C)

BIO12 = Annual Precipitation

BIO15 = Precipitation Seasonality (Coefficient of Variation)

BIO19 = Precipitation of Coldest Quarter

2- Surface moisture and canopy roughness

From QuikScat (QSCAT; http://www.scp.byu.edu/data/Quikscat/SIRv2/qush/World_regions.htm), we obtained monthly raw backscatter measurements that capture attributes related to surface moisture and canopy roughness[4]: Qscat Mean: Annual mean Radar Backscatter of year 2001 (*for spatial distribution of surface moisture and roughness (forest structure*)); less negative means higher backscatter. Unit: Decibel

4- Normalized Difference Vegetation Index

We used the index NDVI (Normalized Difference Vegetation Indices; based on monthly files from the year 2001 of MODIS Data (1km resolution)): NDVI max: Annual maximum NDVI (*for spatial distribution of vegetation density*)

5- Percent Tree Cover

We used the vegetation continuous field[5] product from MODIS (https://lpdaac.usgs.gov/lpdaac/products/modis_overview) as a measure of the percentage of tree cover in 2001.

References

1. Hijmans, R. J., Cameron, S.E., Parra, J. L., Jones, P.G. & Jarvis, A. Very high resolution interpolated climate surfaces for global land areas. *Int. J. Climat.* **25**, 1965-1978 (2005).

2. Nix, H. A biogeographic analysis of Australian elapid snakes. Atlas of Elapid Snakes of Australia. Australian Government Publishing Service (1986).

3. Sehgal, R. N. M. *et al.* Spatially explicit predictions of blood parasites in a widely distributed African rainforest bird. *Proc. R. Soc. Lond. B.* **278**, 1025-1033 (2011).

4. Long, D. G., Drinkwater, M.R., Holt, B., Saatchi, S. & Bertoia, C. Global Ice and Land Climate Studies Using Scatterometer Image Data. *EOS Transactions AGU* **82**, 503 (2001).

5. Hansen, M. C. *et al*. Towards an operational MODIS continuous field of percent tree cover algorithm: examples using AVHRR and MODIS data. *Remote Sensing Envir.* **83**, 303-319 (2002).
